# Clinical and public health management of conjunctivitis in the Israel Defense Forces

**DOI:** 10.1186/s40696-015-0002-3

**Published:** 2015-05-11

**Authors:** Orly Efros, Alon Zahavi, Hagai Levine, Michael Hartal

**Affiliations:** 1 Israel Defense Forces Medical Corps, Military PO Box 02149, Tel Hashomer, Israel; 2 Sackler Faculty of Medicine, Tel Aviv University, Tel Aviv, Israel; 3 Department of Ophthalmology, Rabin Medical Center, Petah Tiqwa, Israel; 4 Hebrew University - Hadassah, Braun School of Public Health and Community Medicine, Ein Kerem, Jerusalem Israel; 5 Department of Military Medicine, Hebrew University, Jerusalem, Israel

**Keywords:** Conjunctivitis, Outbreak, Clinical guidelines, Public health, Military medicine

## Abstract

Acute conjunctivitis is a common diagnosis in the general population, and is especially prevalent among military personnel. Conjunctivitis patients are often contagious, and outbreaks of this infectious condition can cause significant morbidity and may jeopardize military readiness. Early recognition and effective management can prevent additional cases in military units. In this article we review the clinical guidelines and public health policy of the Israel Defense Forces for the management of this important medical condition.

## Introduction

Acute conjunctivitis is a common diagnosis among military personnel, which may lead to loss of training time due to medical unfitness, and in some cases may require patient isolation to prevent the spread of disease among soldiers [1]. Conjunctivitis patients are often contagious, and epidemics of this infectious condition can cause significant morbidity and may jeopardize unit readiness in the military setting. In this article we provide a brief overview of this condition and review the clinical guidelines and public health policy in place in the Israel Defense Forces (IDF) for its management.

### Clinical manifestation

**Figure 1:**
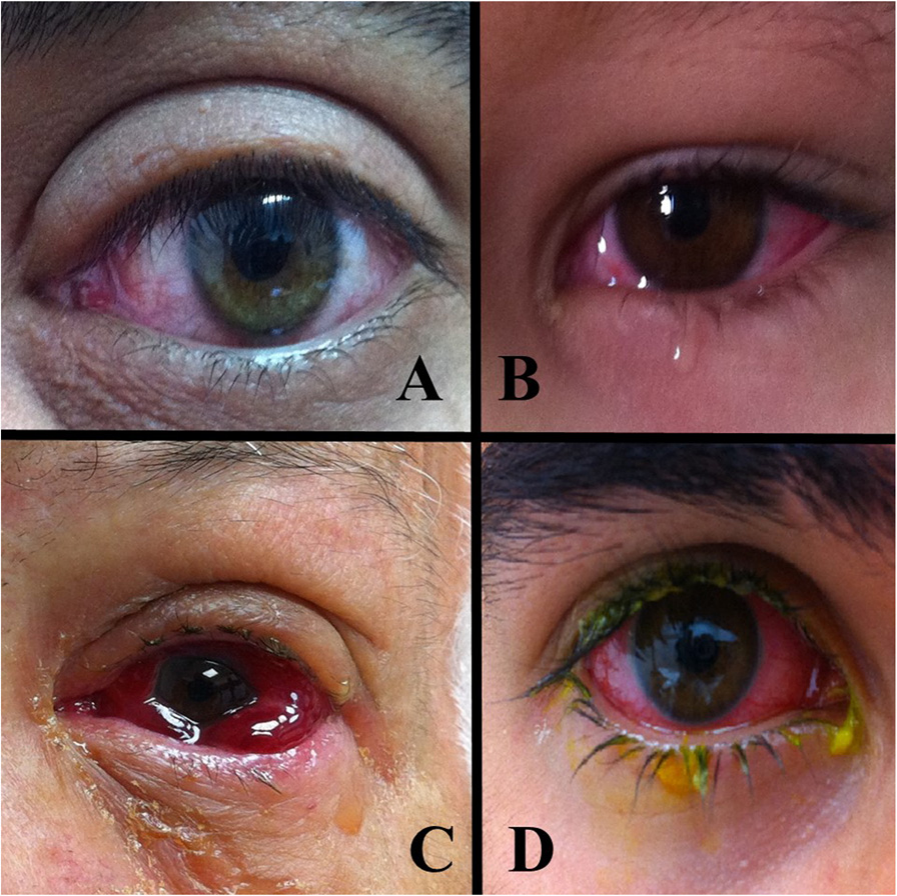
**Clinical manifestations of acute conjunctivitis. A**. Acute viral conjunctivitis. Conjunctival erythema is visible in most cases of viral conjunctivitis. **B**. Acute viral conjunctivitis. Conjunctival erythema, chemosis and tearing are present due to ocular irritation. **C**. Acute conjunctivitis with significant subconjunctival hemorrhage, severe chemosis, and extensive discharge. In addition to lubrication, such cases require topical steroids for symptomatic relief and should be referred for ophthalmic consultation. **D**. Adenoviral conjunctivitis. Examination in an ophthalmology clinic routinely involves fluorescein staining to assess for associated corneal epithelial keratopathy. Tearing, conjunctival hyperemia and chemosis, and secretions are evident.

**Figure 2:**
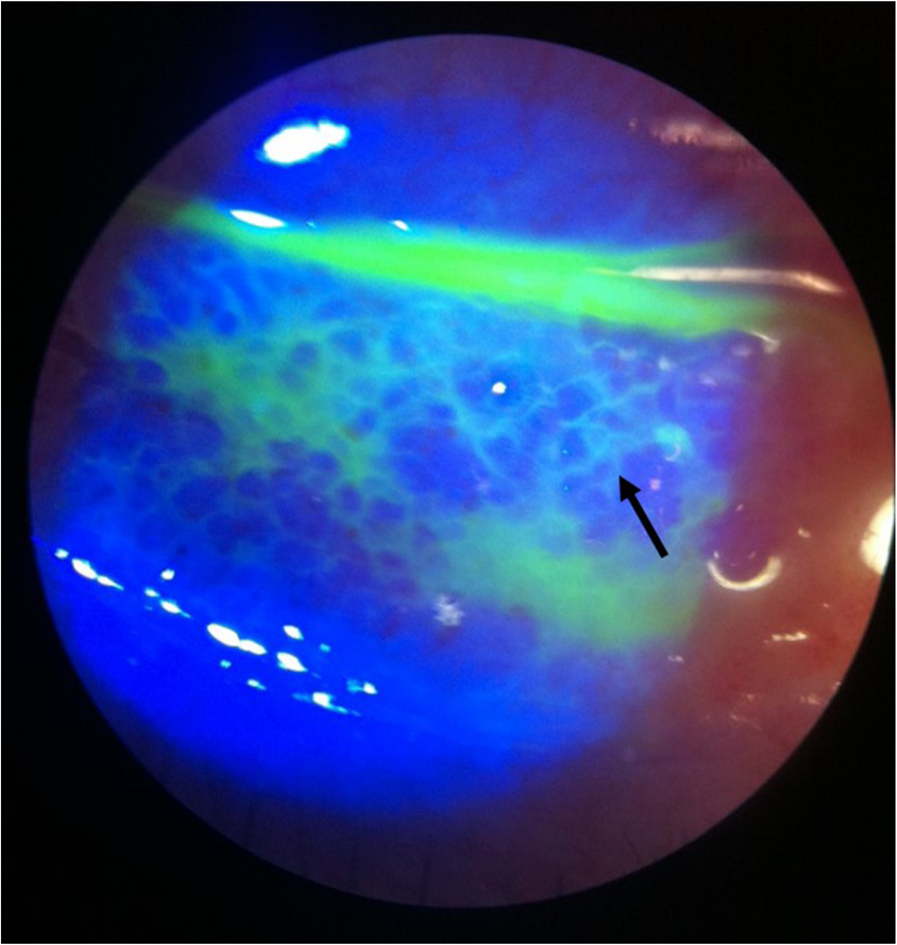
**Slit lamp examination with fluorescein staining in an ophthalmology clinic may also aid in highlighting conjunctival follicular reaction, typical of viral conjunctivitis.**

Clinical manifestations of acute conjunctivitis include tearing, foreign body sensation, redness, and pain. Examination of the infected eye may reveal eyelid edema, discharge or tears (Figure 1A, B), conjunctival hyperemia and chemosis (Figure 1C), and a follicular or papillary reaction on slit lamp examination [2,3] (Figure 2). Corneal findings include superficial punctate keratopathy which may cause pain and photophobia. Physical examination demonstrates pre-auricular and submandibular lymph gland enlargement in approximately 50% of cases [4–6]. Although usually self-limited, infectious conjunctivitis can cause significant morbidity and render the patient unable to perform daily activities.

### Causes

Conjunctivitis can be generally divided into infectious and noninfectious etiologies. Acute conjunctivitis is viral in approximately 80% of cases [4], with adenovirus, the predominant etiologic agent, present in 65-90% of cases [1,4]. Symptoms usually appear following an incubation period of approximately one week, and the patient remains contagious for several weeks [2]. Epidemics have been shown to be a result of person to person transmission [7,2]. Transmission occurs readily through indirect contact with conjunctival secretions, including on door handles and insufficiently chlorinated swimming pools [4,8,9].

Additional viral agents known to cause conjunctivitis include mumps, rubella, influenza and herpetic infections. The latter can often be suspected by the presence of typical cutaneous vesicles in the periorbital region and on the eyelids.

Bacterial infections of the conjunctiva are less common, and are usually caused by *Streptococcus pneumonia*, staphylococcal species, *Haemophilus influenzae* and *Moraxella catarrhalis* [4]. Diagnosis should be confirmed by an ophthalmologist after culturing conjunctival secretions. Conjunctivitis secondary to sexually transmitted diseases such as chlamydia and gonorrhea requires systemic treatment in addition to topical antibiotic therapy, and sexual partners should be informed [2,5].

Bacterial conjunctivitis is transmitted by contact with infected conjunctival discharge. Direct contact with a patient and indirect contact with infected objects such as cosmetics and shared towels contribute to the spread of infection. The infective period begins a few days prior to the appearance of clinical symptoms, and lasts through the entire period of active disease. Furthermore, airborne droplets can be infectious, so that cases are instructed to cover coughs and to avoid sharing drinks and eating from communal dishes.

Noninfectious conjunctivitis has numerous causes. Allergic conjunctivitis usually occurs in patients with a history of allergies and atopic diseases such as asthma and hay fever. Seasonal variation often exists, and clinical symptoms include recurrent episodes of redness and itching. Excessive tearing and eyelid edema are sometimes present. A history of exposure to certain allergens or usage of cosmetic products or topical ocular medications is sometimes elicited [4].

Conjunctivitis caused by intentional application of a foreign object to the eye is called self-inflicted conjunctivitis. This is an uncommon cause of conjunctivitis in both the military and in the civilian population, although several reports have linked this condition to military personnel seeking secondary gain [10,11]. Suspicion should be raised in cases of prolonged conjunctivitis without any apparent cause and non-responsiveness to treatment [12]. Signs of self-inflicted conjunctivitis include excessive subjective complaints in relation to objective clinical findings; repetitive and multiple medical appointments with various physical complaints; concomitant remains of both fresh and dry discharge on the eyelids which might be left intentionally; severity of discharge and edema that exceed conjunctival hyperemia; and involvement of the lower conjunctiva without corneal involvement. However these signs alone are not sufficient for a diagnosis of self-inflicted conjunctivitis and a thorough investigation is warranted [10,11].

Table 1 presents a comparison of the main clinical characteristics of viral, bacterial and self-inflicted conjunctivitis. Various chemical and mechanical injuries can also cause acute conjunctivitis, as can a wide range of clinical conditions uncommon in the military setting [13].

**Table 1:** **Comparison of key clinical characteristics between viral, bacterial and self-inflicted conjunctivitis**

**Clinical characteristics/Etiology**	**Bacterial**	**Viral**	**Self-inflicted**
**Duration**	Can be prolonged without treatment. Treatment hastens recovery	Days to weeks	Can be prolonged. Weeks to months
**Bilateral/unilateral**	Usually spreads to the other eye within days	Usually spreads to the other eye within days	Varies
**Type of discharge**	Mostly purulent discharge	Mostly aqueous, possibly mucoid discharge	Tearing and excessive discharge, fresh and dry purulent discharge on eyelids and periorbital skin
**Swollen lymph glands**	Not common	Common	Not common
**Concomitant signs**	None	Pyrexia , pharyngitis	Emotional or social stress, multiple physical complaints
**Complications**	Uncommon	Uncommon	Uncommon
**Additional findings**	Ocular irritation	Diffuse conjunctival involvement. Foreign body sensation	Mainly involvement of the lower conjunctiva. Discharge and edema are conspicuously prominent in relation to the conjunctival hyperemia
**Response to treatment**	Usually subsides without treatment.	Usually subsides without treatment.	Non-responsive to treatment
	Responds well to antibiotics		
**Epidemiological characteristics**	Contagious. Can lead to an outbreak	Very contagious. Can lead to an outbreak	Rarely the cause of an outbreak. Is usually diagnosed in a single soldier for secondary gain and not in a cluster

### Complications

Complications are relatively rare, and include corneal subepithelial infiltrates as a hypersensitivity reaction to viral antigens. These can cause ocular irritation as well as decreased visual acuity. Sub-conjunctival hemorrhage may cause some discomfort but has no prognostic implications (Figure 1C). Phlyctenules, membranes and pseudomembranes may complicate adenoviral conjunctivitis and cause significant patient discomfort [2,5].

## Review - IDF guidelines and policy

### Primary care

In the IDF, the initial steps in evaluating a patient with acute conjunctivitis include a detailed medical history, a general physical examination and laboratory tests when appropriate. Urgent conditions such as ocular and orbital trauma, foreign bodies, corneal ulcer, keratitis, uveitis, or acute glaucomatous episode often cannot be accurately diagnosed or managed in the primary care clinic. Thus, the diagnosing physician is instructed to identify or rule out clinical signs indicative of these conditions such as acute decrease in vision, proptosis, eye injury or facial injury involving the eye, foreign bodies (including chemical agents), whitish spots on the corneal surface, or significant ocular pain so that the patient can be referred to an ophthalmologist for urgent examination. IDF guidelines require that the medical interview include questions regarding general medical history, family and personal history of ocular diseases and eye infections, duration of ocular complaints, orbital and ocular trauma or penetrating foreign body (including chemical agents), and the use of contact lenses. The physician is instructed to inquire about binocular involvement and the nature of the complaints including ocular irritation, burning sensation, pain, discharge, photophobia, and visual disturbances.

The physical examination begins with assessment of visual acuity using a Snellen chart, pupillary response to light and distance, ocular movements and any elicited pain, and a basic ocular exam including assessment for foreign bodies, periorbital and eyelid redness or edema, ocular protrusion, secretions, conjunctival injection or chemosis, corneal abnormalities, and anterior chamber hyphema or hypopion. Preauricular and submandibular lymph node enlargement is noted if present. The medical history and physical examination are usually sufficient for a diagnosis of acute conjunctivitis and supposition of the possible etiologic agent, obviating the need for additional testing.

**Treatment** is required for symptomatic patients only and no preventive measures are needed for exposed personnel.

In the IDF, primary care doctors, most often battalion and unit physicians, are expected to adequately manage simple cases of acute conjunctivitis. Initial treatment for all cases of conjunctivitis includes cold local compresses, saline solution and tear substitutes.

Since most cases are of viral etiology, treatment is directed towards symptomatic relief and does not shorten the duration of the illness. Topical analgesics may contribute to the development of severe corneal damage if not used properly, and thus they are not indicated for use by primary care physicians in the IDF other than one-time administration in emergency situations, such as during evacuation of soldiers with severe eye pain for expert ophthalmologic evaluation. When secondary bacterial infection is suspected, topical antibiotic treatment such as chloramphenicol 5% ointment is added by the primary care physician. Suspected cases of *N. gonorrhoeae, Chlamidia trachomatis*, and herpetic infections are referred for ophthalmic consultation.

In the IDF, initial treatment of allergic conjunctivitis in the primary care setting includes saline solutions and artificial tears. Topical antihistamines such as tetrahydrozoline drops, or oral chlorpheniramine maleate and decongestants such as pseudoephedrine hydrochloride are also authorized for use by primary care physicians.

Severe cases of conjunctivitis are referred for ophthalmic consultation, and topical nonsteroidal anti-inflammatory drugs and corticosteroids may be considered. These medications, however, are not approved for use in the primary care clinic due to the potential for significant side effects including cataract formation and increased intra ocular pressure leading to glaucoma. It should be noted that in cases of adenoviral conjunctivitis, topical steroids alleviate the symptoms but prolong the infective period. Recurrent cases are referred to an ophthalmologist and occasionally to an allergist.

Long term chronic conjunctivitis unresponsive to therapy raises suspicion regarding self-inflicted conjunctivitis. IDF physicians are directed to attempt identification of the underlying reasons for this self-inflicted injury, mainly through a meticulous patient interview. Clinical policy does not call for direct confrontation with patients suspected of self-inflicted conjunctivitis. Rather, physicians are encouraged to provide counseling and to attempt to resolve underlying issues leading to the factitious injury.

### Preventing transmission of conjunctivitis

Preventing the transmission of communicable conjunctivitis is one of the most important aspects of the clinical management of this condition in the military environment. IDF guidelines designed to prevent transmission within the clinic include the use of disposable gloves during examination, thorough hand washing and disinfection after the examination, disposal of all single-use instruments used during the examination, disinfection of all surfaces the patient has touched, including counters, door handles and chairs, and frequent replacement of multi-use eye drop vials. Soldiers receive detailed guidance regarding personal hygiene practices, including frequent hand washing, proper technique for cleaning secretions with disposable wipes, surface disinfection, avoidance of contact with communal fomites, refraining from sharing soap, towels, glasses, goggles and personal items with other soldiers and avoidance of swimming in communal pools during the communicable period. Isolation of patients is not mandatory, but it can be considered in some cases depending on the clinical condition and the military setting and environment. In addition, clinical monitoring of close contacts is conducted, to facilitate early detection of additional cases and outbreaks.

### Management of outbreaks in the IDF

When the number of diagnosed acute conjunctivitis cases is higher than expected in a given population of soldiers over a defined period of time, the presence of a disease cluster is considered.

When an outbreak is suspected in the IDF, military regulations require rapid reporting to public health officers, in order to enable a timely epidemiological investigation and the implementation of control measures. Reports are initiated by the primary care physician to a regional public health officer, who conducts an initial field investigation in coordination with the reporting physician. Upon confirmation of a potential outbreak, the Epidemiology Section of the Medical Corps Headquarters is also notified, an outbreak record is opened in the central public health database, and guidance is provided as needed. It is possible, however, that some outbreaks of conjunctivitis in the IDF remain unreported due to their short duration, limited scope and mild presentation. Furthermore, the epidemic nature of some clinical clusters may go unnoticed by primary care physicians.

**Figure 3:**
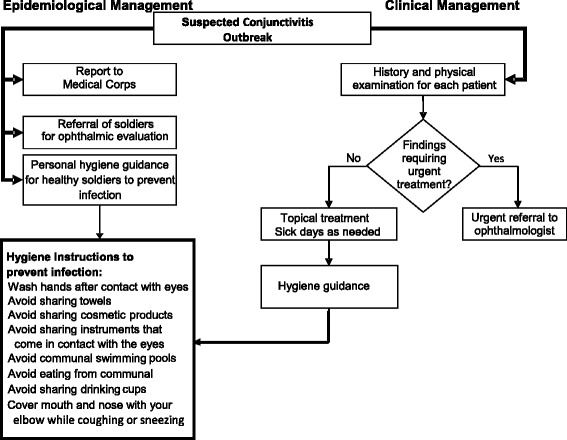
**Suggested management of conjunctivitis outbreak.**

Figure 3 presents IDF guidelines for the management of acute conjunctivitis outbreaks in the primary care setting. These guidelines include both individual patient guidance and general instructions for close contacts in the unit, and are designed to interrupt transmission and prevent recurrence of the disease. Outbreak management starts with verifying that the clinical guidelines described above (interview, examination, treatment, personal hygiene) have been implemented. Unit commanders are briefed early on regarding the medical and operational ramifications of the outbreak, and active case finding is utilized to detect cases that have not presented for medical treatment. A liberal approach to granting off-base sick leave is often prudent in minimizing person-to-person transmission within the unit, and medical clearance is prerequisite for return to duty. Although most cases of conjunctivitis are self-limited, cultures are taken from a subset of patients in order to establish the etiologic agent and guide outbreak management.

## Conclusions

In this manuscript we reviewed the IDF’s policy and guidelines for the clinical and public health management of conjunctivitis, which can have significant impact on military personnel health. Conjunctivitis is a common clinical condition in the military setting. While most cases are sporadic, epidemics are not a rare occurrence. Most cases of infectious conjunctivitis are of a viral etiology and are highly contagious, increasing the risk of rapid spread and high infection rates in the military setting. Prompt diagnosis and effective treatment in individual conjunctivitis cases and informed management of outbreaks are paramount in reducing the scope of morbidity within the unit, preserving military readiness.
